# Physiological Role of β-Catenin/TCF Signaling in Neurons of the Adult Brain

**DOI:** 10.1007/s11064-013-0980-9

**Published:** 2013-02-02

**Authors:** Marta B. Wisniewska

**Affiliations:** 1 Laboratory of Neurodegeneration, International Institute of Molecular and Cell Biology, ul. Ks. Trojdena 4, 02-109 Warsaw, Poland; 2 Present Address: Centre of New Technologies, University of Warsaw, Zwirki i Wigury 93, 02-089 Warsaw, Poland

**Keywords:** Wnt, LEF1/TCF, Brain, Neurogenesis, NMDA, Thalamus

## Abstract

Wnt/β-catenin pathway, the effectors of which are transcription factors of the LEF1/TCF family, is primarily associated with development. Strikingly, however, some of the genes of the pathway are schizophrenia susceptibility genes, and the proteins that are often mutated in neurodegenerative diseases have the ability to regulate β-catenin levels. If impairment of this pathway indeed leads to these pathologies, then it likely plays a physiological role in the adult brain. This review provides an overview of the current knowledge on this subject. The involvement of β-catenin and LEF1/TCF factors in adult neurogenesis, synaptic plasticity, and the function of thalamic neurons are discussed. The data are still very preliminary and often based on circumstantial or indirect evidence. Further research might help to understand the etiology of the aforementioned pathologies.

## Introduction

### β-Catenin, its Functions and Regulation

β-Catenin has diverse functions in a cell that are mediated by its interactions with other proteins. β-Catenin stabilizes cell–cell junctions by anchoring cell adhesion molecules (i.e., Cadherins) in the actin cytoskeleton, activates the LEF1/TCF family of transcription factors, and is involved in the amplification and separation of centrosomes [1]. β-Catenin is abundant at the plasma membrane, but its cytoplasmic/nuclear pool is tightly regulated.

**Fig. 1:**
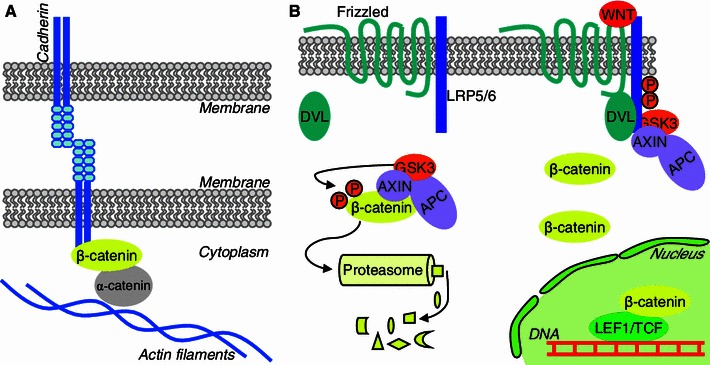
β-Catenin plays two main functions in the cell. **a** β-Catenin interacts with Cadherins at the internal site of the membrane and links them to the actin cytoskeleton together with α-catenin. **b** Cytoplasmic β-catenin plays an effector role in the canonical Wnt pathway. In the absence of WNT ligands, β-catenin is phosphorylated by GSK3α/β and subsequently degraded in the proteasome. Upon Wnt stimulation of a Frizzled receptor, Dishevelled (DVL) recruits AXIN to the LRP5/6 co-receptor, β-catenin is stabilized, enters the nucleus, and activates LEF1/TCF transcription factors

The majority of β-catenin is bound to trans-membrane Cadherin proteins. This complex, connected to actin via α-catenin, constitutes adherens junctions by which cells attach firmly to one another [1] (Fig. 1a). In neurons, membranous β-catenin plays an additional role in the synapse. Catherin-β-catenin adhesion complexes form symmetric synaptic junctions that border synaptic active zones [2]. Synaptic β-catenin regulates the size and localization of vesicle clusters by interacting with PDZ proteins at presynaptic sites [3–5]. It also targets some scaffolding molecules at postsynaptic sites [6, 7] and influences excitatory postsynaptic strength [8]. Detailed reviews on the role of β-catenin and Cadherins in synapses can be found elsewhere [9–11].

The cytoplasmic fraction of β-catenin is regulated by phosphorylation in a destruction complex that is composed of glycogen synthase kinase 3α (GSK3α) or GSK3β and two scaffolding proteins, AXIN and adematous polyposis coli (APC) [12]. The phosphorylation of β-catenin by GSK3α/β creates a signal for its rapid degradation in the proteasome. A classic way that β-catenin is activated is the inhibition of GSK3α/β in the canonical Wnt signaling pathway, which is also called the Wnt/β-catenin pathway (Fig. 1b). Signal transduction is initiated by the interaction between secreted glycoproteins, Wnts (mammals have 19 Wnt proteins), and Frizzled receptors (mammals have 10), in a paracrine or autocrine fashion. Upon the binding of Wnt to Frizzled receptors and low-density lipoprotein receptor-related protein 5 or 6 (LRP5/6) co-receptors, the destruction complex is inhibited, promoting the accumulation of β-catenin and enabling its translocation to the nucleus. Thereafter, together with LEF1/TCF transcription factors, β-catenin acts as a transcriptional activator. Four LEF1/TCF transcription factors have been identified: LEF1, TCF7, TCF7L1, and TCF7L2 (the last three are also known as TCF1, TCF3, and TCF4, respectively). These factors belong to the high mobility group family and bind the same DNA motif; however, they can mediate diverse functions by activating different sets of genes [13]. The canonical Wnt pathway is one of the principal developmental pathways. It is involved in body axis specification, morphogenesis, and stem cell proliferation and differentiation [14]. Its role in forebrain development, including neurogenesis, is very well established [15–18]. New data suggest that it can also be active in adult neurogenic niches and in postmitotic neurons, which will be discussed below.

**Fig. 2:**
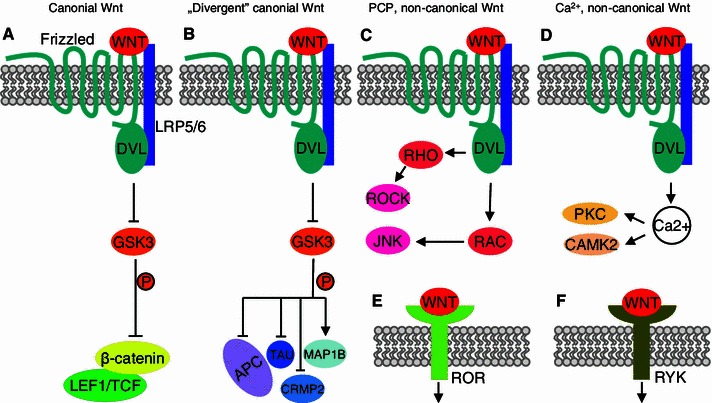
Wnt ligands induce diverse Wnt pathways. **a, b** Wnt ligands bind Frizzled receptors and activate canonical Wnt signaling. The inhibition of GSK3α/β in the canonical Wnt pathway can lead to the activation of β-catenin or influence other targets of GSK3α/β. These two possibilities can be called the Wnt/β-catenin pathway and “divergent canonical” Wnt pathway, respectively. **c** Other WNT molecules and Frizzled receptors can activate planar cell polarity (PCP) signaling, in which downstream messenger are ROCK and JNK kinases. **d** WNT5a can regulate intracellular level of Ca^2+^, which is involved in many cellular signalings and can inhibit β-catenin/TCF signaling. **e, f** WNT molecules can also bind to the receptor tyrosine kinases (RTK) ROR and RYK. The Wnt/RYK pathway regulates neuronal differentiation

The inhibition of GSK3α/β by Wnt signaling, β-catenin stabilization and LEF1/TCF transcription factor activation are closely linked events, but they are not inseparable. Canonical signaling (Fig. 2a) can diverge from the classic pathway downstream of GSK3α/β (Fig. 2b). For example, in neurons, the inhibition of GSK3α/β by Wnt can lead to a decrease in microtubule-associated protein 1B (MAP1B) phosphorylation by this kinase, resulting in microtubule stabilization [19, 20]. β-Catenin can also be stabilized by Wnt-independent mechanisms. The present review refers to any mechanism of β-catenin stabilization and activation of LEF1/TCF transcription factors as β-catenin/TCF signaling.

Several excellent recent reviews have been published on the role of synaptic β-catenin and “divergent canonical” (i.e., Wnt/GSK3) (Fig. 1b) and noncanonical Wnt (diverse Wnt/Frizzled-initiated signaling independent of GSK3α/β inhibition) pathways (Fig. 2c–f) in synaptic plasticity [10, 21]. This review does not discuss these topics but rather focuses on the involvement of β-catenin/TCF signaling in neuronal function, which is a relatively new and enigmatic topic.

### β-Catenin/TCF Signaling and Adult Brain Pathologies

GSK3α/β has been implicated in affective and psychotic disorders and neurodegeneration. Unclear, however, is whether GSK3α/β acts through β-catenin in these brain pathologies. Nevertheless, some links have been made between β-catenin/TCF signaling and schizophrenia, bipolar disorder, Alzheimer’s disease, Parkinson’s disease, and Huntington’s disease (AD, PD, and HD, respectively).

For the latter diseases, some proteins that are encoded by genes that are mutated in neurodegenerative disorders (e.g., Parkin in PD, Huntingtin in HD, and presenilins in AD) have been shown to regulate β-catenin levels [22–25]. In the case of psychotic and mood disorders, scientists’ attention turned to the potential involvement of β-catenin with the discovery that lithium ions, a classic and effective medication for bipolar disorder, can inhibit GSK3α/β [26, 27]. Interestingly, the *FZD3*, *GSK*-*3β*, *DKK4*, *APC*, and *TCF7L2* genes that encode Wnt pathway components have been shown to be associated with susceptibility to schizophrenia [28–33]. Moreover, anxiety-like phenotypes were observed in *Tcf7l2* haplo-insufficient mice [34], whereas the brain-specific overexpression of β-catenin exerted mood-stabilizing-like effects in standard models of mania and depression [35], suggesting a connection between dysregulated β-catenin/TCF signaling and psychiatric disorders.

Until now, the involvement of β-catenin in brain pathologies is only an attractive hypothesis. Before it can be either confirmed or rejected, more research should be conducted to determine the physiological role of the β-catenin/TCF signaling pathway in neurons in the adult brain. The present review summarizes the current knowledge on this subject.

## β-Catenin Signaling in Adult Neurogenesis

Canonical Wnt/β-catenin signaling plays a crucial role in development in general. Specifically, it is involved in multiple aspects of central nervous system development [15, 17, 18, 36, 37]. β-Catenin has been shown to regulate the self-renewal of neural progenitor cells and neuronal differentiation in the developing neocortical ventricular zone [15, 16, 38, 39], subcortical areas of the telencephalon [40], and ventral midbrain [41]. This raises the issue of whether canonical Wnt signaling is also involved in adult neurogenesis.

### Adult Neurogenic Niches

In the adult brain, neurogenesis persists in the subventricular zone (SVZ) of the lateral ventricle in the cortex and subgranular zone (SGZ) in the hippocampus [42]. Two types of adult neural stem cells or neural progenitor cells (aNPCs) have been characterized in these germinal zones: primary progenitors that are slowly dividing cells and intermediate progenitors that intensively proliferate. Both types of aNPCs express the SOX2 marker. The primary progenitors, glial fibrillary acidic protein (GFAP)-positive cells, have a radial glia-like phenotype and are named Type 1 progenitors in the SVZ and B progenitors in the SGZ. They have the capacity to generate neurons, astrocytes, and oligodendrocytes. The intermediate progenitors that express proneuronal factor MASH1+ are called Type 2 or C progenitors, respectively. During neurogenesis, the intermediate progenitors adopt a neuronal fate, defined among others by TUJ1 expression, and give rise to proliferating and migrating neuroblasts that are doublecortin (DCX) and polysialic acid-neural cell adhesion molecule (PSA–NCAM)-positive. DCX and PSA–NCAM are also markers of new neurons that additionally express the pan-neuronal marker NeuN.

The major adult neurogenic niche is the SVZ, from which neuroblasts migrate along the rostral migratory stream (RMS) to the olfactory bulbs and differentiate into dopaminergic and γ-aminobutyric acid (GABA) interneurons. The neuroblasts from the SGZ migrate only a short distance and give rise to granular glutamatergic neurons that integrate in the dentate gyrus of the hippocampus. The progression from aNPCs to differentiated neurons involves progenitor cell maintenance and proliferation, a cell-fate decision, neuroblast proliferation and migration, neuronal maturation, and finally the integration of new neurons into the neuronal circuitry. All of the stages of neurogenesis occur through the implementation of internal genetic and epigenetic programs and are under the control of microenvironmental factors [43–45]. One of the implicated signaling pathways is Wnt/β-catenin.

### Activity of the Canonical Wnt Pathway in the Adult Neurogenic Niche

Several components of the canonical Wnt pathway have been described in the adult neurogenic niche. The *Wnt3*, *Wnt5a*, *Wnt7a*, *Wnt8b*, and Frizzled receptor (*Fz*) 1, 2, and 9 genes are expressed in the SGZ of the dentate gyrus and hilus [46, 47], whereas *Wnt1*, *Wnt5a*, *Wnt7a*, *Wnt7b*, *Fz3*, *Fz4*, *Fz7*, *Fz8*, and *Fz9* are expressed in cortical aNPCs in the SVZ or along the RMS [46, 48]. This suggests the possibility that the pathway can be activated in these brain areas in adults.

The hallmark of pathway activity and its effector phase is the nuclear localization of β-catenin. Unfortunately, to the author’s knowledge, no convincing nuclear immunostaining of β-catenin has been demonstrated in the hippocampus, along the lateral ventricles, or in the olfactory bulbs [49–52]. Yet it should be kept in mind that the sensitivity of immunodetection is limited, and the detection of nuclear β-catenin is particularly challenging because of its abundant amounts at the cell membranes. Another concern is the possible action of β-catenin as a transcription activator in the adult neurogenic niche, with low expression of LEF1/TCF genes. Considering the extant in situ hybridization data [46, 53] and Allen Brain Atlas (http://mouse.brain-map.org; accessed November 15, 2012), no apparent or reproducible expression of these genes is found in the SGZ, SVZ, or RMS, with the exception perhaps of weak *Tcf7* expression along the RMS [46]. Notably, however, canonical Wnt signaling is not expected to be constantly “on” during aNPC proliferation and differentiation but rather to be transiently activated in precisely determined places and at specific times. Therefore, its activity can be difficult to observe in vivo under physiological conditions.

To overcome this obstacle, several reporter systems have been used to visualize the activity of the canonical Wnt pathway using LacZ staining or fluorescent protein. In BATGAL mice, reporter activity in the SGZ of the hippocampus was observed in proliferating cells, including neuroblasts, but not in newly generated neurons [47]. Consistent with these results, intense activity of the reporter in ins-TOPGAL reporter mice was found in aNPCs in the SGZ [54]. However, only 10 % of DCX+ cells, representing neuron-committed cells and new neurons, displayed reporter activity. This result suggests downregulation of Wnt/β-catenin signaling at the neuroblast stage, whereas the results in BATGAL mice imply later inhibition. The same contradiction can be observed in the SVZ when comparing BATGAL and AXIN2-d2EGFP reporter mice. In both mice, reporter activity in the SVZ was detected in aNPCs [55, 56], but it was also detected in some DCX+ cells in BATGAL mice [56]. Despite these discrepancies, the overall picture of Wnt/β-catenin pathway activity in the adult neurogenic niche appears to be consistent. The expression of some Wnt molecules and their receptors can be found in the niches, and the activity of β-catenin/TCFs complexes can be indirectly observed, at least in aNPCs.

### Role of Wnt/β-Catenin Signaling in Adult Neurogenesis in the Hippocampus

The first study on the involvement of the canonical Wnt pathway in adult neurogenesis concluded that β-catenin/TCF signaling is a principal positive regulator of neurogenesis in the adult hippocampus [47]. Signaling appeared to be activated by agents secreted by hippocampal astrocytes, probably Wnt3A, which was shown to be expressed in close proximity to the SGZ. When the Wnt/β-catenin pathway was inhibited in aNPCs co-cultured with hippocampal astrocytes, by the WNT inhibitor sFRP2/3 or ectopic expression of dominant-negative (dn) *Lef1*, the percentage of neuronal lineage cells (DCX+, TUJ1+, or MAP2+) decreased. Moreover, the ectopic expression of *Wnt3a* in aNPC monocultures enhanced the determination of neuronal fate and stimulated the proliferation of neuroblasts [47]. In situ experiments, in which lentiviruses that carried *dnWnt* or *Wnt3* were stereotactically injected into the mouse dentate gyrus of the hippocampus, confirmed the positive influence of the signaling on neuroblast proliferation [47]. β-Catenin was also shown to be involved in the maturation of hippocampal neurons. The conditional loss of β-catenin in newborn neurons of the SGZ led to dendritic malformation, again suggesting its positive role in neurogenesis at a later stage [57]. Nonetheless, this latter study did not determine whether the defects were attributable to a loss of β-catenin’s transcriptional role or its function in cell adhesion.

An opposing scenario was proposed, based on the research on *Gfap*–*Cre*-driven APC knockout (KO) mice, in which β-catenin was stabilized in primary aNPCs, regardless of canonical Wnt signaling status [58]. The number of neuronal-committed cells (DCX+ or BrdU+/NEUN+) dramatically decreased in the SGZ in these mice, suggesting that canonical Wnt signaling is a potent inhibitor, not activator, of neuronal differentiation. In another study, consistent with the previous one, elimination of baseline Wnt activity by the Wnt inhibitor Frizzled8-CRD-Fc or *Axin2* overexpression increased the percentage of neuronal-lineage cells under differentiation conditions in vitro [59]. Care should be taken, however, when drawing conclusions about the negative impact of Wnt/β-catenin or β-catenin/TCF signaling on adult neurogenesis based on these studies because the specific involvement of β-catenin was not tested. Thus, other effectors of the Wnt pathway may have mediated this anti-neurogenic effect.

### Role of Wnt/β-Catenin Signaling in Adult Neurogenesis in the SVZ and Olfactory Bulbs

Investigations on the role of β-catenin signaling in SVZ neurogenesis have also generated conflicting results and an even more complex view of Wnt involvement. Several studies demonstrated a clear mitogenic effect of β-catenin signaling in primary and intermediate progenitors (i.e., B and C cells). The activation of β-catenin by *Wnt3a*, *Wnt5a*, or *Wnt7a* [60, 61] and the ectopic expression of a stable β-catenin form, i.e., Δ90β-catenin, increased the proliferation of neural progenitors in vitro, whereas blockade of the canonical Wnt pathway by a Wnt antagonist or ectopic expression of *Axin* had an opposite effect [61]. The above experiments were performed under strong mitogenic stimulation provided by epidermal and fibroblast growth factors (FGF2 and EGF), thus should be interpreted with caution. But nonetheless, their results were confirmed in vivo. The intracranial lentiviral delivery of *Axin* decreased proliferation in adult neurogenic niches, whereas the ectopic expression of Δ90β-catenin enhanced proliferation in the SVZ, at least in B progenitors (GFAP+) [61]. In agreement with these experiments, the delivery of Δ90β-catenin into mitotic cells in the SVZ by stereotactic retroviral injection increased the number of C progenitors (MASH+) [55]. Conversely, the inhibition of Wnt/β-catenin signaling the retroviral delivery of *Dkk1*, which encodes an LRP inhibitor, decreased this number. Thus, according to the above studies, Wnt/β-catenin signaling promotes the expansion of the neural progenitor pool.

In sharp contrast to the above analyses are results of other in vivo studies, which were performed on a transgenic mouse line that expressed another stabilized form of β-catenin, β-catenin^Ex3^, in an inducible manner [56]. The stabilization of β-catenin was achieved by Tamoxifen-dependent and tissue specific deletion of β-catenin exon 3. The mice in which β-catenin^Ex3^ expression was induced in adulthood in Nestin-positive cells (type B and C progenitors), exhibited an approximately two-fold reduction of proliferation in the SVZ 1 week after the induction. Explaining this inconsistency is difficult when considering the different ages of the mice used in these studies, which varied from 1 to 2 months. The mode of β-catenin stabilization also unlikely mattered because the Δ90β-catenin and β-catenin^Ex3^ forms are not substantially different.

Likewise, vague is the role of β-catenin in the differentiation of neurons in the SVZ and along the RMS. aNPC cultures exhibited an approximately twofold increase in the percentage of mature (MAP2+) neurons under differentiation conditions when treated with WNT3a or WNT5a [60]. This resulted from an increase in the neuronal fate choice of the progenitors and was not caused by an expansion of neuroblasts. The same occurrence (i.e., a twofold increase in the proportion of newly generated [PSA-NCAM+] neurons) was observed in vivo in the SVZ upon the induction of β-catenin^Ex3^ [56], which was accompanied by an increase in *Lef1* expression, a well-known target gene of β-catenin/TCF.

In contrast, the cell-specific deletion of APC, leading to the accumulation of β-catenin in intermediate progenitors (MASH+) in the SVZ, dramatically decreased the number of neuroblasts (DCX+) in vivo, implying an essential impairment of neurogenesis at the stage of neuroblast generation [58]. However, in *APC* KO differentiation cultures, neuronal lineage cells (TUJ1+) displayed abnormal morphology with no neurites and did not express markers of newly generated neurons, DCX or PSA-NCAM. This suggests that APC deletion did not prevent the induction of neuronal fate but rather disturbed proper differentiation and maturation. Moreover, whether this effect was β-catenin-dependent was not determined. APC, in addition to regulating β-catenin levels, can bind to microtubules and influence axon growth; thus, the described phenotype could be, at least partially, attributable to this action of APC [62].

### Does the Wnt/β-Catenin Pathway Determine the Self-Renewal or Differentiation of aNPCs?

In summary, most of the studies presented herein indicate a positive effect of Wnt/β-catenin signaling on aNPC proliferation [55, 60, 61] and determination of neuronal fate [47, 56, 60]. Additionally, Wnt/β-catenin signaling promoted the proliferation of hippocampal aNPCs [47]. Nevertheless, research that demonstrated the opposite (e.g., an inhibitory effect of β-catenin on aNPC proliferation in the SVZ [56]) cannot be ignored. β-Catenin may activate different, even opposing, genetic programs, depending on the particular context (e.g., the availability of specific partners) [63, 64]. Therefore, β-catenin/TCF signaling can likely exert both positive and negative effects on the maintenance of neuronal progenitors and neuronal differentiation, what could explain the discrepancies between different studies. This possibility will be discussed in the next chapter.

Although direct proof of the involvement of gene regulation by β-catenin/TCF complexes in adult neurogenesis is mostly lacking in the discussed works, a multitude of indirect evidence makes its role in this process widely accepted. Nonetheless, one should be cautious when drawing conclusions. For example, although treatment with WNT3a or pharmacological inhibition of GSK3α/β increased the neuronal differentiation of NPCs isolated from neonatal mouse cortex [48], the activation of β-catenin/TCF was detected only in a small fraction of these cells, and most of them expressed neither the neural progenitor marker Nestin nor marker of neuronal committed cells TUJ1. Thus, mechanisms other than activation of β-catenin might be responsible for the promotion of neuronal differentiation, such as cross-talk of the Wnt/GSK3 pathway with the Notch pathway [48, 65].

### β-Catenin Activators and Partners Might Modify the Outcome of β-Catenin/TCF Signaling in Neuronal Differentiation

Canonical Wnt signaling is known to be context-dependent, [66]. This means that the precise time and manner of Wnt pathway activation and specific cellular microenvironment affect the final outcome of the signaling. Efforts to determine the actual profile of Wnt pathway components, including activators, inhibitors, and mediators, and the occurrence of associated factors under specific physiological conditions might help explain its role at different stages of neurogenesis.

A good example of research that is moving in this direction is the analysis of WNT7A and TLX (NR2E1) relationships during the proliferation of aNPCs [61]. *Wnt7a* and *Tlx* are both expressed in proliferating but not differentiating aNPCs in vitro [61]. TLX, a transcription factor, is a known regulator of aNPC self-renewal [47, 67], whereas the *Wnt7a* gene can be activated by TLX [61]. *Tlx* silencing or *Tlx* KO caused a deficiency of aNPC proliferation (in vitro or in vivo in the SVZ), which could be rescued by WNT7A treatment or expression of the active form of β-catenin [61]. This suggests that TLX promotes aNPC proliferation by inducing production of WNT7A, which then activates β-catenin in an autocrine manner.

**Fig. 3:**
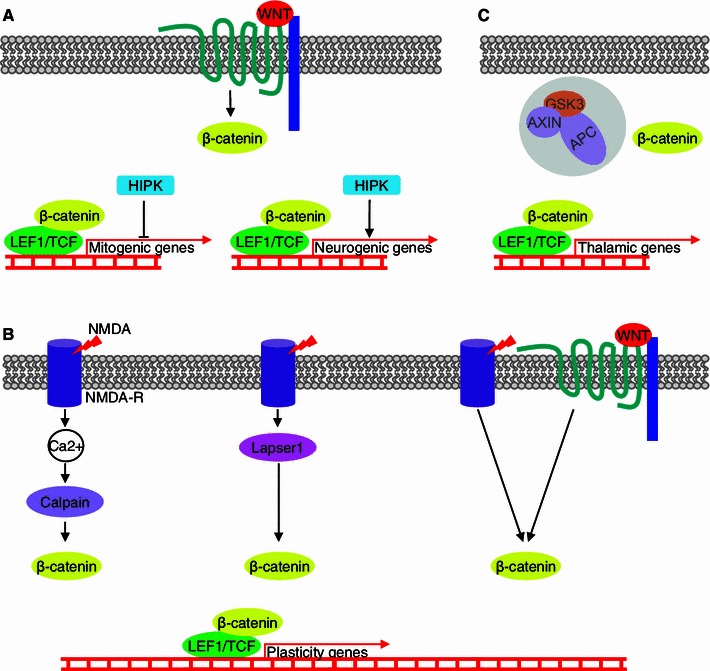
Possible roles of β-catenin in neurons in the adult brain. **a** A hypothetical model of mitogenic and neurogenic roles of β-catenin/TCF complexes during adult neurogenesis, modulated by HIPK1 [56, 61]. **b** Three hypothetical scenarios of NMDA-dependent nuclear translocation of β-catenin, involving Calpain [85], Lapser1 [86], and Wnt [84]. **c** Constant activity of β-catenin/TCF complexes in thalamic neurons [89]

A protein that might be involved in modulating the outcome of canonical Wnt signaling activation is HIPK1 from the HIPK family of seronine/threonine kinases. HIPK proteins can interact with β-catenin and activate or repress β-catenin/TCF target genes [56, 68]. *Hipk1* is expressed in embryonic and adult neurogenic niches [56]. Interestingly, alterations of HIPK1 and β-catenin levels in embryonic neurogenic niches influenced the balance between cell proliferation and differentiation, the latter estimated based on the number of neural progenitors (RC2+ cells) and new neuronal-committed cells (TUJ1+) [56]. This suggests that HIPK1 can switch the β-catenin-dependent program from proliferation to differentiation. Because the level of *Hipk1* expression strongly increases in primary (B-type) progenitors in the SVZ in adults, the hypothesized role of HIPK1 is to modify the outcome of β-catenin signaling in aNPCs toward the inhibition of stem cell renewal and promotion of neuronal differentiation (Fig. 3a) [56].

### β-Catenin/TCF Target Genes in Neuronal Differentiation

To understand the precise mechanism of action of canonical Wnt signaling during adult neurogenesis, determining its actual target genes during both the clonal expansion and differentiation of neuronal progenitors is necessary. Many of the known β-catenin/TCF targets encode proteins that are involved in cell cycle progression [69], for example the *Cyclin D1* gene, making the signaling a potent activator of cell proliferation. Whether these genes are indeed activated in proliferating neuronal progenitors by β-catenin is not known. With regard to neuronal differentiation, cell-type specific target genes are expected to be activated by canonical Wnt. To date, three direct targets of β-catenin/TCF have been described in differentiating aNPCs. One of these is the *Ink4a* gene that encodes a general inhibitor of cell proliferation [70], and the other two, *Neurod1* and *Prox1*, encode proteins that are transcription factors specifically involved in neuronal differentiation [71–73].

The *Ink4a* promoter has been previously shown to have a LEF1/TCF motif [74] and be inhibited by β-catenin in a melanoma cell line [75]. In neurons, β-catenin appears to activate the *Ink4a* gene in aNPCs in vitro but only in cooperation with HIPK1, which is mentioned in the previous chapter [56]. In turn, activation of the *Neurod1* promoter by β-catenin in aNPCs depends on the overlapping SOX/TCF binding site [76]. SOX2 is a repressor of neuronal differentiation [77, 78], and β-catenin is likely able to reverse the repression by removing SOX2 from the *Neurod1* promoter. Finally, chromatin immunoprecipitation assay performed on aNPC showed association of β-catenin with the enhancer region of *Prox1*, which is specifically expressed in the dentate gyrus in adults [71]. Many more genes can likely be regulated by β-catenin/TCF during adult neurogenesis. This was indicated by an in silico study that revealed the substantial and highly significant enrichment of putative LEF/TCF target genes in the genes that encode proteins involved in neuronal differentiation [79].

## Activation of β-Catenin and Synaptic Plasticity

### NMDA-Dependent Nuclear Translocation of β-Catenin

Intensive synaptogenesis occurs during early development and infancy; nevertheless, new synapses are formed in the brain throughout life. Depending on neuronal activity, synapses disappear, are stabilized, are weakened, or are strengthened [80]. These phenomena, which are referred to as synaptic plasticity, allow the refinement of neuronal circuits in the mature brain and provide a basis for learning and memory formation. Synaptic activity is associated with signal transduction both locally in the synapse and at the cell-wide level. An example of local changes is dephosphorylation of β-catenin at Tyr654 and its subsequent translocation from dendritic shafts to dendritic spines, followed by the binding to Cadherins [81]. Long-lasting changes in synapses, however, require the activation of gene expression [82]. Such intracellular responses to neuronal excitation are regulated by signaling cascades that are initiated mainly by Ca^2+^ influx through the ionotropic receptors of neurotransmitters, such as *N*-methyl-d-aspartate (NMDA) [83]. The classic pathway in this context is the activation of the transcription factor cyclic adenosine monophosphate response element binding protein (CREB) mediated by Ca^2+^ and calcium/calmodulin-dependent kinase IV [82].

Several years ago, β-catenin was hypothesized to be used to transduce some synaptic activity-dependent signals into the nucleus (Fig. 3b) [41]. The hypothesis was based on the observation that tetanic stimulation of dentate gyrus neurons in hippocampal slices induced the release of WNT3a and increased the nuclear accumulation of β-catenin by approximately 20 % [84]. The activation of β-catenin was NMDA-dependent because it was blocked by the NMDA receptor antagonist APV. Wnt signaling was also required for the accumulation of β-catenin, which was demonstrated by treatment with Frizzled8-CRD-Fc, a WNT inhibitor. Importantly, activation of the canonical Wnt pathway during stimulation had a positive impact on long-term potentiation. Its magnitude decreased when Wnt signaling was blocked by Frizzled8-Fc or an anti-WNT3a antibody and increased when it was additionally activated by recombinant WNT3a or LiCl [84].

Although two other groups of researchers confirmed the nuclear localization of β-catenin in neurons upon stimulation of NMDA receptors in rat hippocampal cells in culture, they proposed different mechanisms of β-catenin accumulation and concluded that the Wnt pathway was not involved [85, 86]. In the first study, β-catenin was found to be cleaved at the *N*-terminus after stimulation. Only a *C*-terminus-specific antibody could detect β-catenin in the nuclei of stimulated hippocampal neurons, implying that exclisively the truncated pool translocates [85]. The truncation of β-catenin was effectuated by Calpain, a Ca^2+^-activated protease. Application of the Calpain inhibitor MDL28170 abolished not only cleavage but also translocation in a Wnt-independent manner, indicating a new mechanism of β-catenin regulation in stimulated neurons. The authors proposed that the cleavage led to the accumulation of β-catenin by preventing its degradation because the truncated isoform could not be phosphorylated by GSK3α/β and consequently could not be targeted to the proteasome (Fig. 3b). However, the other study detected nuclear β-catenin by both *C*-terminus- and *N*-terminus-specific antibodies, conflicting with the cleavage hypothesis. They proposed that Lapser1, which belongs to the Fezzin family of postsynaptic density proteins [87], can interact with β-catenin [88] and is involved in the redistribution of β-catenin after synaptic stimulation [86] (Fig. 3b). Lapser1 was found to shuttle to the nucleus upon treatment with NMDA or glutamate but not α-amino-3-hydroxy-5-methyl-4-isoxazolepropionic acid (AMPA) or K^+^, similar to β-catenin [86]. The authors claimed that although Lapser1 silencing did not abolish the nuclear import of β-catenin, it impaired its later export. Some doubts, however, are raised by the fact that high concentrations of β-catenin in cell bodies and nuclei were visible even in non-stimulated neurons, which was contradictory to what was shown by others in primary cultures of hippocampal neurons [85, 89].

The only in vivo evidence for the neuronal excitation-dependent activation of β-catenin was derived from a study performed in TOPGAL mice that were subjected to a novelty exploration test [85]. The number of β-galactosidase-positive neurons in the CA3 field of the hippocampus increased, indirectly indicating the activation of β-catenin/TCF-dependent genes. Treatment with MDL28170 abolished this effect, confirming the involvement of Calpain. Further studies are necessary to confirm the nuclear translocation of β-catenin and determine its actual importance in synaptic plasticity and learning.

### Possible Target Genes of β-Catenin/TCF in Excited Neurons

An expected effect of the nuclear translocation of β-catenin in excited neurons is the activation of plasticity-related genes. A set of known β-catenin/TCF targets (*Cd44*, *Ccnd1*, *Cldn1*, *Dll1*, *Fas*, *Wisp2*, *Fosl1*, and *Myc*) were found to be upregulated after tetanic stimulation or glutamate treatment [84–86]. Interestingly, an in silico genome-wide analysis revealed significant enrichment of putative LEF1/TCF targets in neuronal genes that encode voltage-gated channels and synaptic proteins, including structural proteins, neurotransmitter receptors, and proteins associated with synaptic vesicles [79]. These genes are good candidates for β-catenin/TCF targets in glutamate-stimulated neurons when considering their possible impact on synaptic plasticity, provided that they are actually activated by the treatment, which is currently unknown.

## Role of β-Catenin in the Adult Thalamus

### Constant Activity of β-Catenin in Thalamic Neurons

β-Catenin is an intracellular signaling effector that supposedly undergoes nuclear translocation upon activation. Surprisingly, however, the constant nuclear localization of β-catenin was observed in neurons in subcortical structures that are involved in the integration of sensory information, including the thalamus, epithalamus, pretectum, periaqueductal grey, and superior colliculus [50, 52]. Interestingly, the accumulation of β-catenin was accompanied by high levels of TCF7L2 and LEF1 proteins [52]. LEF1 was also detected in the entorhinal cortex, and TCF7L2 was detected in the inferior colliculus, but β-catenin was not detected in the cellular nuclei in these regions [52].

What causes β-catenin to accumulate in the nuclei of these neurons? This phenomenon is apparently cell-autonomous because growing thalamic neurons in dissociated primary cultures without glial cells did not abolish the nuclear localization of β-catenin [89]. β-Catenin accumulation appears to be independent of Wnt pathway activation. The inhibition of Wnt signal transmission by treatment with DKK1, an inhibitor of LRP5/6 co-receptors, or ectopic expression of a dominant-negative form of Disheveled, a mediator of Wnt signaling that acts downstream of the Frizzled receptor, did not affect the nuclear accumulation of β-catenin [89]. The accumulation instead seemed to result from low levels of proteins that constitute the β-catenin destruction complex: GSK3α/β, AXIN1, and APC [89] (Fig. 3c). Indeed, degradation of the cytoplasmic fraction of β-catenin is less effective in thalamic neurons than in cortical or hippocampal neurons.

### Genetic Program Activated by β-Catenin in the Thalamus

Gene profiling in the forebrain revealed that voltage-gated ion channel and neurotransmitter receptor genes, which have at least two LEF1/TCF motifs in their putative regulatory sequences, are preferentially expressed in the thalamus [79]. A loss-of-function experiment in primary thalamic cultures by the ectopic expression of *Axin2*, and immunoprecipitation of forebrain chromatin with an anti-β-catenin antibody provided evidence of the direct regulation of at least *Cacna1g*, *Kcna6* (voltage-gated channel genes), *Gabra2* (GABA receptor gene), and *Calb2* (which encodes a calcium buffer, Calretinin) in the thalamus [79]. The *Cacna1g* promoter in particular was shown to be activated by LEF1 and β-catenin in a luciferase assay and to bind LEF1 in a footprinting test [49], confirming that it is a genuine target of β-catenin and LEF1/TCF factors. *Cacna1g* encodes the Cav3.1 subunit of so-called T-type channels that are critical for the function of thalamocortical relay neurons. T-type channels underlie the principal electrical property of these neurons, i.e., the intrinsic possibility to fire in two modes [90]. This feature enables the modification of the format of information that is sent to the cortex and is believed to regulate sleep/wakefulness and awareness [91]. Importantly, an additional activation of β-catenin enhanced T-type current in thalamic neurons in culture [49]. The constant nuclear localization of β-catenin and LEF1/TCF transcription factors in thalamic neurons in adults, together with the regulation of crucial thalamic genes by these factors, suggests that β-catenin/TCF might maintain molecular identity of thalamic neurons.

## Future Directions

Knowledge about the role of canonical Wnt signaling effectors in the adult brain is still relatively superficial and inconclusive. Many studies have focused on the activation or inhibition of the canonical Wnt pathway while assuming concomitant changes in nuclear β-catenin activity and the availability of LEF1/TCF factors. In turn, when directly targeting β-catenin, research faces a major difficulty when attempting to delineate its adhesive and nuclear functions in a given cellular or physiological process. Wnt research suffers from insufficient tools to inhibit the nuclear canonical Wnt pathway and a lack of sophisticated animal models. The hope is that such tools and models will be soon be developed and implemented in fundamental and application neuroscience.
